# Quiescent Systemic Lupus Erythematosus Manifesting with Thrombotic Thrombocytopenic Purpura and Acute Renal Failure: A Case Report and Short Literature Review

**DOI:** 10.31138/mjr.32.4.358

**Published:** 2021-12-27

**Authors:** Konstantinos Melissaropoulos, Panagiotis Georgiou

**Affiliations:** 1 Department of Rheumatology, Agios Andreas General Hospital, Patras, Greece

**Keywords:** lupus, thrombotic microangiopathy, thrombotic thrombocytopenic purpura

## Abstract

Systemic lupus erythematosus (SLE) is a multisystem autoimmune disease, and haematologic manifestations are part of its spectrum. Herein, we report a case of a patient with a long-standing diagnosis of SLE, presenting with thrombotic thrombocytopenic purpura (TTP) and acute renal failure, without co-existent clinical and laboratory markers of disease activity, causing diagnostic questions. A short literature review concerning TTP and SLE is also presented. TTP is a rare syndrome of thrombotic microangiopathy, which represents a medical urgency and carries significant morbidity and mortality if left untreated. SLE has been correlated with the occurrence of TTP, often with atypical presentation and worse prognosis.

## INTRODUCTION

Systemic lupus erythematosus (SLE) is the prototypic autoimmune disease affecting multiple systems and exhibiting remarkable heterogeneity. The disease pathophysiology is complex, but loss of self-tolerance, autoantibodies production, and defective clearance of immune- complexes are considered key processes.^1^ Haematologic manifestations are part of the disease spectrum, presenting commonly as cytopenias and haemostasis dysregulation.^2^ Peripheral destruction through circulating autoantibodies is the underlying mechanism in a variety of disorders, such as in cases of immune-mediated leukopenia, thrombocytopenia, and autoimmune haemolytic anaemia. Rare blood manifestations have also been reported and SLE-associated thrombotic thrombocytopenic purpura (sTTP) belongs to them.

Thrombotic thrombocytopenic purpura (TTP) is a hematologic disease and represents a medical emergency. It manifests with features of thrombotic microangiopathy consisting of microangiopathic haemolytic anaemia, thrombocytopenia, and histological features of capillary and small vessel thrombosis. Fever, neurological, and renal involvement may coexist. Searching for schistocytes in the peripheral blood smear is vital for an early diagnosis in case of suspicion. The differential diagnosis of a patient with thrombotic microangiopathy includes several primary diseases; however, secondary causes associated with common conditions such as infection and cancer should be kept in mind. Among the primary causes, TTP is a well-characterized disease with a distinct pathophysiologic mechanism, involving large multimers of the von Willebrand factor (vWF) caused by the deficiency of a vWF-cleaving protease named ADAMTS-13. This is often correlated with an autoantibody induced mechanism of inhibition in acquired forms of the disease.^3^ Protease activity <10% is highly supportive of TTP diagnosis.^4,5^ However, atypical features of sTTP are reported in the literature, and the role of ADAMTS-13 is not as clear as in idiopathic TTP cases, implying a more complex underlying pathogenesis.^6^ Herein, we present a case of a patient with SLE without evident disease activity who developed a thrombotic microangiopathy syndrome with acute renal failure diagnosed and successfully treated as TTP.

## CASE

A 64-year-old female patient with known SLE diagnosis presented in the emergency department with fatigue and headache. Because the initial laboratory assessment revealed anaemia, thrombocytopenia, and elevated creatinine, the patient was admitted to the hospital.

The patient’s medical history included a first SLE diagnosis 20 years ago, when she developed severe immune-mediated thrombocytopenia with antinuclear and anti-Smith autoantibodies present in her serum, responding to high dose steroid treatment. For several years, the patient was achieving remission on hydroxychloroquine and low-dose steroids. The year before her recent admission, we were puzzled by an acute establishment of sensorineural left ear hearing loss, refractory to high dose steroids. Cyclophosphamide was added to the patient’s treatment. Minimal improvement was recorded after five cyclophosphamide pulses, with the last one received one month before her recent admission to the hospital.

During her recent hospitalization, the patient’s initial evaluation revealed a markedly increased lactate dehydrogenase (LDH), elevated indirect bilirubin, and the presence of schistocytes in the peripheral blood smear, compatible with a thrombotic microangiopathy syndrome. Direct Coombs test was negative, fibrinogen and complement components were in a normal range and the erythrocyte sedimentation rate was moderately increased to a value of 43 mm/h. The clinical evaluation did not reveal skin rash, arthritis, or other signs of active lupus. Fundoscopy for malignant hypertension, blood cultures, and computed tomography scanning were negative for associated conditions like infection or cancer. Serum creatinine was > 6 mg/dl, with mild proteinuria (<1gr/24h) and without signs of active urine sediment indicative of immune glomerulonephritis. On the contrary, the sediment evaluation revealed “muddy brown” granular casts indicative of acute tubular necrosis. Based on these findings, the patient was diagnosed with TTP in the context of SLE. The patient was promptly treated with intravenous steroids (1 gr methylprednisolone daily for three consecutive days followed by 1mg/kg of body weight daily). Concurrently, she started plasma exchange therapies and haemodialysis. Renal function quickly improved to normal creatinine levels; however, the hematologic parameters were not stabilized 20 days after treatment initiation. Four weekly rituximab infusions, at a dose of 375mg/m^2^ of body surface area each, were added to the patient’s treatment regimen with significant laboratory improvement and the patient managed to exit the hospital.

Approximately four weeks after exiting the hospital and rituximab scheme completion, the patient was on 8mg methylprednisolone *per os* and laboratory parameters were normal. Unfortunately, she developed a lung infection, and the patient was readmitted to the hospital due to dyspnoea. She initially failed to respond to broad-spectrum antibiotics. Taking into account the patient’s immunocompromised status, appropriate treatment was added for opportunistic infections, including trimethoprim-sulfamethoxazole in high doses. She slowly improved and managed to exit the hospital and continue treatment at home. Until now, the patient remains in a stable condition with normal range laboratory values and without clinical signs of active lupus.

## LITERATURE REVIEW

We performed a literature search using the MEDLINE database to identify cases of SLE associated thrombotic microangiopathy diagnosed as or resembling TTP. An additional manual search in the bibliography of retrieved articles was also performed. In our study were included all case series with five or more cases from 1998 until 2020, written in English language and concerning adults. The study by Musio et al.^7^ published in 1998 was an extensive review of all published cases until then. Additional literature reviews have been published since then aiming to identify all cases of sTTP.^8–10^ We chose to perform a concise narrative review to explore qualitative features of TTP like disease in the context of SLE, treatment and mortality trends through the years.

Nine case series fulfilling our criteria were identified, including 181 patients.^11–19^ Their clinical characteristics, treatment and mortality rate are presented along with the patients from the review by Musio et al.^7^ (**Table 1**). Most sTTP cases reported (85.5%) already had a diagnosis of SLE. A concurrent diagnosis of these two entities can occur, however, and the clinician should be vigilant for features of SLE in all new cases of TTP. Unlike the review by Musio et al.,^7^ where active SLE was found in 37% of the cases reported, the majority of the rest of the patients presented here had active lupus, based on clinical and serologic markers. In this context, it is not strange that most authors reported renal dysfunction in their patients. In the case series by Aleem et al.,^12^ where biopsy data were available for all patients, lupus nephritis was evident in all specimens, with 50% also exhibiting signs of thrombotic microangiopathy in renal vasculature. Regarding treatment schemes, steroids and plasma exchange therapy were commonly used. Cytotoxic treatment, in the form of cyclophosphamide and vincristine mainly, was also a therapeutic choice especially in refractory disease and/or a pre-existing SLE diagnosis. We observed a less common use of rituximab among these patients. However, a more frequent use was recorded in the latest years. Infection rate ranged between 8% and 54%, showing that this is a significant complication. Mortality ranged from 0 to 62.5%, with no clear trend through the years. This could be explained by the heterogeneity of patients recorded, including age, lupus disease duration and infection rate. Data about ADAMTS-13 were provided only in few studies. In the study by Merayo-Chalico et al.,^18^ mean plasmatic activity was 41%. In the most recent article by Yue et al.,^19^ only patients with severe ADAMTS-13 deficiency and the presence of ADAMTS-13 inhibitor were included, exhibiting a favourable response. Interestingly, Matsuyama et al.^16^ reported the presence of two phenotypes of thrombotic microangiopathy related to SLE and other connective tissue diseases. A minor population (23.4% of SLE cases) was found to have severely deficient ADAMTS-13 activity with neutralizing autoantibodies, resembling typical acquired idiopathic TTP. The rest of their cases had moderate to mild enzyme deficiency, or even normal ADAMTS-13. The latter population had worse prognosis.

**Table 1.** Clinical characteristics, treatment, and prognosis in SLE related TTP-like disease.

**Reference**	**Patients (n)**	**Time of TTP diagnosis in relation to SLE diagnosis**	**Active SLE at TTP diagnosis**	**Treatment**		**Infection**	**Mortality**
Musio et al.^7^	41	Precedent (15%)	37%	GC (100%)	CYC (15%)	NS	34%
		Concurrent (12%)		PE (68%)	VINC (17%)		
		Subsequent (73%)		PI (12%)	IVIG (7%)		
Dold et al.^11^	15	Subsequent (100%)	NS	GC (100%)	IMM (27%)	NS	0
			(mSL 15.3)	PE (40%)	IVIG (7%)		
Aleem et al.^12^	6	Concurrent (67%)	100%	GC (100%)	PI (17%)	NS	0
		Subsequent (33%)		PE (83%)	IMM (17%)		
Majithia et al.^13^	5	Subsequent (100%)	100%	GC (100%)	CYC (80%)	20%	0
				PE (100%)	VINC (60%)		
Kwok et al.^14^	26	Concurrent (12%)	NS	GC (88%)	IMM (42%)	31%	46%
		Subsequent (88%)	(mSL 14)	PE (92%)	IVIG (35%)		
Letchumanan et al.^15^	8	Concurrent (25%)	100%	GC (100%)	VINC (25%)	12.5%	62.5%
		Subsequent (75%)		PE (100%)	RTX (37.5%)		
				CYC (75%)	IVIG (37.5%)		
Matsuyama et al.^16^	64	NS	NS	GC (91%)	PI (27%)	NS	26%
				PE (70%)	IMM (31%)		
Chen et al.^17^	25	Concurrent (8%)	88%	GC (100%)	RTX (4%)	8% ^a^	52%
		Subsequent (92%)		PE (64%)	IVIG (4%)		
				CYC (4%)	IMM (28%)		
Merayo-Chalico et al.^18^	22	Concurrent (27%)	NS	GC (95%)	IMM (33%)	54%	20%
		Subsequent (73%)	(mSL 12.3)	PE (87%)	IVIG (8%)		
				CYC (16%)	RTX (4%)		
				VINC (29%)			
Yue et al.^19^	10	NS	100%	GC (100%)	RTX (20%)	NS	0
				PE (70%)	IVIG (50%)		
				CYC (80%)	IMM (10%)		

**Abbreviations**: SLE, systemic lupus erythematosus; TTP, thrombotic thrombocytopenic purpura; NS, not specified; GC, glucocorticoids; PE, plasma exchange; PI, plasma infusion; CYC, cyclophosphamide; VINC, vincristine; RTX, rituximab; IMM, immunosuppressive drugs including azathioprine, mycophenolate or not specified; IVIG, intravenous immunoglobulin; mSL, mean SLEDAI (Systemic Lupus Erythematosus Disease Activity Index).

a Authors report concomitant infection possibly triggering TTP like disease in 64% of their cases, not included in the post treatment infection rate presented here.

## DISCUSSION

We chose to present this case to highlight a rare lupus manifestation, which appeared in our patient although she was stable regarding her autoimmune disease. In fact, she was at the end of a monthly intravenous cyclophosphamide treatment scheme for sensorineural hearing loss at the time of TTP diagnosis, a cytotoxic therapy also used to treat sTTP. Clinical and laboratory parameters showed lupus remission before and during her recent hospitalization. Even though most case-series correlate sTTP occurrence with high lupus disease activity,^14,15,18^ there are reports of non-active lupus presenting with TTP.^7,20^ This highlights the need for increased vigilance in SLE presenting with thrombotic microangiopathy, even in patients with long-standing remission. TTP is a diagnosis where treatment should be implemented promptly. This requires a high level of suspicion and quick differential diagnosis of other conditions manifesting with thrombotic microangiopathy, such as infection/disseminated intravascular coagulation, cancer and pregnancy morbidity.^4^

Renal failure, which was evident in our patient, posed significant diagnostic questions, as idiopathic TTP cases rarely manifest with severe renal involvement. The presence of lupus nephritis was the first scenario to be excluded since the patient had an established lupus diagnosis. Lupus nephritis has been implicated in micro-angiopathic haemolytic anaemia initially diagnosed and treated as TTP^21^ and is often found in biopsy specimens of such patients.^7,12^ In our case, the urine sediment did not show signs of glomerulonephritis. Instead, it was suggestive of acute tubular necrosis. The patient indeed improved very quickly with supportive measures, with normalisation of serum creatinine and proteinuria. Therapy in patients with suspected TTP needs to be implemented as soon as possible. Plasmapheresis is the cornerstone of treatment, having changed TTP prognosis in recent years. Immunosuppression is commonly added in the form of corticosteroids, while rituximab is the recommended add-on treatment nowadays in severe and refractory cases.^22^ Regarding lupus, sTTP should be treated in the same way even though many case series report the need for more intense immunosuppression when lupus activity signs are present.

Data in the literature regarding morbidity and mortality provide non-clear assumptions. It seems that sTTP is associated with prolonged hospitalisation,^18^ with some case series showing increased mortality in comparison with idiopathic cases^15^ and others showing the opposite.18,19 We should consider when evaluating those conclusions that this is a rare condition and thus the number of cases is small, as well as the fact that diagnostic methods and treatment have changed through the years. In older case series, broad immunosuppressants such as cyclophosphamide were frequently used, while rituximab tends to be common practice nowadays. In addition, contrary to recent case series,^19^ which have used ADAMTS-13 activity as an essential component of diagnosis, data about the enzyme activity are not always available in older studies. Even when those data exist, there seems to be a less severe deficiency in sTTP, rendering the role of ADAMTS-13 in these cases controversial.^6^ The pathophysiology underlying thrombotic microangiopathy occurrence in the context of SLE appears to be far more complicated than the straightforward lack of ADAMTS-13 activity in idiopathic TTP and incorporates aspects of true lupus active disease including vascular inflammation, as well as immune mediated non inflammatory vWF multimerisation and platelet aggregation fuelled by a crescendo of autoimmunity often seen in lupus.^23^ Considering these, the cases recruited in these studies probably lack homogeneity, and perhaps future studies should include stricter criteria for TTP diagnosis in the context of SLE. Unfortunately, assays evaluating ADAMTS-13 activity and the presence of antibodies against this protease are not always available, and this was also true in our case. SLE is associated with an increased burden of comorbidities and chronic organ damage. Drug-related toxicity and infections are a major cause of morbidity and death in these patients. In a recent meta-analysis, patients with SLE exhibited an approximately three-fold pooled risk for overall severe infection compared to the general population, including opportunistic infections.^24^ In a retrospective case series of a single centre assessing the occurrence of TTP in SLE, researchers found that infection was the only independent risk factor for mortality.^14^ It should always be kept in mind however, that a pre-existing infection could present with thrombotic microangiopathy and thus mimic TTP in these patients, as probably happened in the case series by Chen et al.^17^ In our case, severe immunosuppression with cyclophosphamide, rituximab and glucocorticoids led to the patient’s hospitalization with pneumonia after her hematologic parameters had normalized. We believe that she sustained an opportunistic infection with *Pneumocystis jirovecii*, considering that she only responded to high doses of trimethoprim-sulfamethoxazole.

In conclusion, sTTP is a rare manifestation in SLE, can occur both in active and non-active lupus, and needs prompt diagnosis and treatment. The clinician should always exclude other conditions that also present with thrombotic microangiopathy and common symptoms and signs such as fever, neurologic, and renal involvement. ADAMTS-13 activity assays can offer substantial help in establishing a correct diagnosis, even though this protease’s role in sTTP is not as clear as in idiopathic TTP.
